# The potential role of genus *Treponema* in carcinogenesis with a focus on oral squamous cell carcinoma: a scoping review of the evidence

**DOI:** 10.1186/s12903-025-07118-4

**Published:** 2025-11-26

**Authors:** Pratibha Gopalkrishna, Krishnananda Prabhu, Lakshmi Puzhankara, Madhurya Kedlaya, Somasish Ghosh Dastidar, Monica Charlotte Solomon, Thokur Sreepathy Murali

**Affiliations:** 1 Department of Periodontology, Manipal College of Dental Sciences, Manipal, Manipal Academy of Higher Education, Manipal, India; 2 Department of Biochemistry, Kasturba Medical College, Manipal, Manipal Academy of Higher Education, Manipal, India; 3 Centre for Molecular Neurosciences, Kasturba Medical College, Manipal, Manipal Academy of Higher Education, Manipal, India; 4 Department of Oral Pathology, Manipal College of Dental Sciences, Manipal, Manipal Academy of Higher Education, Manipal, India; 5 Department of Public Health Genomics, Manipal School of Life Sciences, Manipal, Manipal Academy of Higher Education, Manipal, India

**Keywords:** Dysbiosis, Microbiome, Carcinoma, Spirochaetes/spirochetes, Treponema denticola, Oral microbiome, Dentilisin (Td-CTLP), Outer membrane vesicles (OMVs)

## Abstract

**Background:**

Current concepts suggest that a dysbiotic environment can promote ‘oral carcinomas.’ Microbiome studies on the oral cavity indicate changes in bacterial disposition in this condition. Yet, few focus on a lesser-known species, *Treponema denticola*, a motile periodontal pathogen, in addressing concerns related to oral carcinogenesis. Clinical studies find an enrichment of the *Treponema* genus in sites with oral cancer. Other research designs hint at *Treponema denticola* possessing both direct and indirect mechanisms to perpetrate damage in oral cancer.

**Methods:**

We followed the Joanna Briggs Institute (JBI) methodology for this scoping review. The population, Concept and Context (PCC) were as follows: Population: adults with carcinomas in general/OSCC; Concept: *T. denticola/Treponema* and virulence factors; Context: Presence of *T. denticola/Treponema* and virulence factors in oral cavity tissues/fluids / or demonstrating role in carcinogenesis. The terms ‘Adult OR population OR patient AND Treponema denticola OR T. denticola OR spirochete* OR treponema* AND oral cancer OR OSCC OR oral squamous cell carcinoma OR carcinoma OR metastasis OR epithelial-mesenchymal transition OR Oral cancer initiation,* promotion*,* progression’* were adapted and searched across four different databases, retrieving all material published in English till 26 August 2024.

**Results:**

Sixty-six articles were included in the scoping review following a full-text search, including 35 clinical studies, 21 reviews, 3 database studies, 4 in vitro studies, and 2 animal studies. Approximately 54% of the clinical studies found spirochetes or *Trep*o*nema* (genus/species) or its virulence factor abundant at cancer sites. Animal models also demonstrate the impact of *Treponema denticola* on tumour progression.

**Conclusions:**

The genus *Treponema* and/or its virulence factors are detected in some oral carcinoma samples, indicating a possible association with advanced stages or deeper invasion. Future research can focus on its ability to induce malignant transformation and explore its potential as a candidate biomarker of oral carcinoma deserving validation.

**Supplementary Information:**

The online version contains supplementary material available at 10.1186/s12903-025-07118-4.

## Background

Oral squamous cell carcinoma (OSCC) often demonstrates poor prognosis, high mortality rates and poor overall survival statistics, afflicting a considerably large proportion of the population in the South Asian region. Traditionally attributed to abusive habits like smoking and excessive alcohol intake, other risk factors for oral carcinomas like poor oral hygiene and a diet rich in processed meat and low in fruits and vegetables [1] are currently being explored. Concepts proposed recently for OSCC pathogenesis consider the dysbiosis of the oral environment and the involvement of keystone pathogenic organisms. Studies have shown an association of specific groups of bacteria in patients with OSCC [2, 3].

Yet, disparity exists in the observed microbial profile. While there is agreement that microflora implicated in periodontal disease are present at distant cancer sites, the changes in the quantity and quality of these bacteria contributing to carcinomatous lesions remain a grey area. *Fusobacterium nucleatum (F. nucleatum)* and *Porphyromonas gingivalis (P. gingivalis)* are notably prominent in the gastrointestinal [4, 5] and oral carcinomas [6, 7].

Animal studies that focused on *Treponema denticola (T. denticola)* in tumour development implicated the organism in increasing tumour size and metastasis. *T. denticola* is often allied with *P. gingivalis* in the oral cavity [8]. *Treponema* as a species is less consistent, and often genus-level presence is observed compared to *F. nucleatum* and *P. gingivalis*. The association of this lesser-known spirochete with oral carcinoma and its pathogenesis forms the focus of this scoping review.

## Main text

*Treponema* species are part of the biofilm on the tooth surface and are considered commensals in the oral cavity. However, their pathogenic potential becomes evident in conditions such as necrotising gingival and periodontal lesions, endodontic infections, and abscesses. Oral *Treponema* now includes up to 49 subspecies, thanks to recent advances in genome sequencing techniques [9].

Factors such as the ‘major sheath protein’ and ‘dentilisin’ facilitate tissue colonisation and epithelial cell invasion. The outer membrane vesicles contribute to their long-range action. Saglie et al. [10] observed spirochetes to be present even between closely adhering junctional epithelial cells of the periodontium.

While studies on periodontitis have focused on the role of *T. denticola*, its effects on carcinogenesis are relatively undetermined. Studies suggest its presence at distant cancer sites, such as in colorectal cancer [11]. Advances in microbiome research have renewed interest in these previously unculturable bacteria and their potential association with oral carcinomas. Our scoping review aims to gather comprehensive data from all available information sources on this seemingly low-profile oral spirochete and its role in carcinogenesis in general and OSCC in particular. Therefore, we included all oral microbiome studies and studies on the role of this organism in carcinogenesis and OSCC, while excluding studies solely on periodontitis and those that did not include *T. denticola*. Periodontitis itself represents not only a risk factor but also a strong confounder when examining the association between *Treponema denticola* and OSCC. The interpretation of available evidence is influenced by how well individual studies addressed these potential confounders. Most of the included studies have adjusted for the occurrence of periodontal disease through the study design. While certain investigations have adjusted for factors such as periodontal status, oral hygiene, tobacco and alcohol use, and in some cases human papillomavirus (HPV) infection, these adjustments were not applied uniformly across all studies.

Against this background, our review critically examines the potential role of *T. denticola* in carcinogenesis, especially OSCC, by synthesising existing evidence and identifying areas where future research can facilitate more efficient identification of the possible contributory or etiological role of this pathogen in carcinogenesis.

### Methods

The JBI methodology for scoping reviews was followed for the preparation of this paper [12].

## Review question

Population: adults with carcinomas in general/OSCC;

Concept: *T. denticola/Treponema* and virulence factors;

Context: Presence of *T. denticola/Treponema* and virulence factors in oral cavity tissues/fluids/or demonstrating role in carcinogenesis.

Focused question: Does the presence of *T. denticola* facilitate initiation, promotion and progression of carcinomas in general and OSCC in particular in adults?

We explored databases of Scopus, Embase, PubMed and Web of Science. We also looked at the Google Scholar search engine. The keywords utilised for the search in the various databases were ‘*Adult* OR *population* OR *patient* AND *Treponema denticola* OR *T. denticola* OR spirochete* OR treponema* AND *oral cancer* OR *OSCC* OR *oral squamous cell carcinoma* OR *carcinoma* OR *metastasis* OR *epithelial-mesenchymal transition* OR *Oral cancer initiation*,* promotion*,* progression*.’ Our review considered all studies published in English only up to August 26, 2024.

## Inclusion and exclusion criteria

### Participants

Studies with adult patients having OSCC or carcinomas and healthy controls were included.

### Studies

We selected articles in English using search terms: oral cancer/oral carcinoma/OSCC/carcinoma and *Treponema denticola* (Supplementary Material 2). We excluded studies which did not analyse *spirochaetal* bacteria. References on *Treponema* that did not mention carcinoma, oral cancer or OSCC were excluded. Studies that included *Treponema* species but were focused on periodontal disease were also excluded.

### Concept

Identified as a motile spirochete in dark field microscopy, *Treponema denticola* is an obligate, Gram-negative anaerobe that can cause chronic inflammation and modulate the immune response. It also secretes proteases that degrade local tissues, creating an environment conducive to Oral carcinomas. The prevalence of *T. denticola* in carcinoma in general and OSCC in particular, and the mechanisms by which it contributes towards the etiopathogenesis of OSCC, are less explored. Hence, the review examines the role of *T. denticola* in carcinogenesis in general, and with a focus on OSCC.

### Context

*T. denticola* is found in elevated levels in patients with poor oral hygiene and periodontitis. This organism may likely be related to carcinogenesis or OSCC, as poor oral hygiene and periodontitis are considered potential risk factors for the condition. Hence, if the participants have carcinoma, OSCC and the presence of bacterial class, spirochetes, and virulence factors in oral cavity tissues/fluids/or demonstrate a role in carcinogenesis, they have been included in the review. We excluded studies on adults with diseases other than oral squamous cell carcinoma.

### Types of sources

A variety of study designs were evaluated, including preclinical animal studies and observational studies, such as case-control and cross-sectional study designs. We included narrative and systematic reviews that met the inclusion criteria.

### Search strategy

An initial PubMed search was conducted to retrieve articles on the topic (Supplementary Material 3). We used MeSH (Medical Subject Headings) terms in the search for related articles. Utilising those words, we adopted a detailed search strategy for the other databases to generate a list of references. Using a data extraction tool to collect relevant information, we selected articles published in English only.

### Source of evidence selection

Consistent with JBI guidance, we sought all relevant sources of evidence to map the field, including human observational studies, interventional trials (if any), in vitro and animal studies, database re-analyses, and prior reviews/grey literature. The evidence types were charted and reported as separate categories; no quantitative synthesis was attempted.

**Fig. 1:**
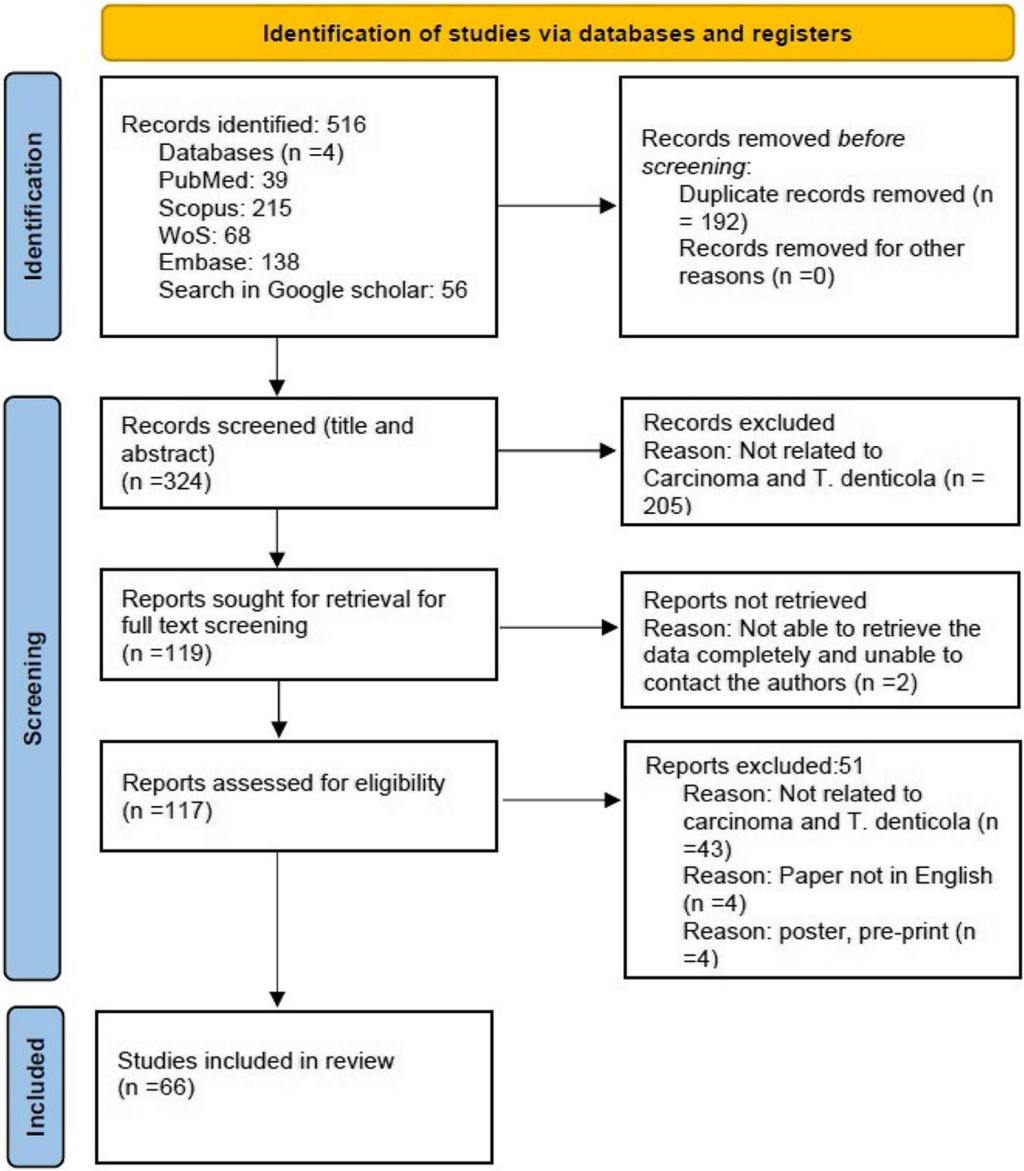
PRISMA (Scoping review) flow chart

The search in PubMed, SCOPUS, EMBASE, Web of Science and Google Scholar identified citations, which were subsequently collated and uploaded into the Mendeley reference manager to remove duplicates. Three reviewers, PG, LP and MK, screened the titles and abstracts independently to assess eligibility according to the inclusion criteria. The full texts of the retrieved articles were identified during this screening, followed by reviewer assessment for compliance with the inclusion criteria. We excluded those sources of evidence that did not meet the decided criteria. The reviewers resolved their disagreements through discussion. The flow diagram (Fig. 1) presents the results of the search and the inclusion process based on the PRISMA-ScR format (Preferred Reporting Items for Systematic Reviews and Meta-analysis extension for scoping reviews) [13].

### Data extraction

These reviewers collectively decided on the data extraction strategy, which included participant details, concept, context, methods adopted and findings relevant to the review question. The data extraction table included details such as author, year, country, sample size, sample analysed, method of detection, mechanism of action of *T. denticola* in carcinogenesis, virulence factor studied, inference of the study, and associated cancer type.

## Results

### Data analysis and presentation

The database search yielded 516 results for the search terms, with about 192 duplicate papers. After removing the duplicates, we performed title and abstract screening of 324 papers. We eliminated 205 papers and did the full-text screening of 119 papers. Two papers were non-retrievable and hence excluded. Fifty-one papers were excluded after full-text screening to include 66 papers in this scoping review (Fig. 1): 35 observational studies, 21 reviews, three database studies, four in-vitro studies, two animal studies and a letter to the editor. Two dissertations on *Treponema denticola* were also included in the review. The included papers demonstrate a global distribution: 19 from the USA, 13 papers from China, 11 papers from India, 6 from Finland, three from Korea, two each from Japan, Romania, Sweden and Taiwan, and one each from Canada, Iran, Columbia, Poland, UK and Australia (Supplementary Material 1 - Table).

#### Clinical studies

We included 16 cross-sectional, 15 case-control and 4 cohort studies, comprising 1095 samples from cross-sectional studies and 700 from the cohort studies (Table 1). In the case-control studies, there were 661 cases and 714 controls.

**Table 1:** Summary of clinical studies

S. No	Author, Year, Country	Sample size	Sample type	Method of detection	Mechanism of action of T. denticola in OSCC pathogenesis	Virulence factor studied	Inference of the study	Associated Cancer Type
1	Cai et al. 2024, Cross sectional study, Hong Kong, China [14]	90	Tumour and adjacent normal tissues	Host genome-wide transcriptome and DNA CpG methylation	-	-	*Treponema medium* was among the seven bacterial species enriched in OSCC patients along with *Fusobacterium nucleatum*,* Peptostreptococcus stomatis*,* Gemella morbillorum*,* Catonella morbi*,* Peptoanaerobacter yurli and Peptococcus simiae*	OSCC
2	Li et al. 2023, Cross-sectional study, China [15]	112 OSCC and 20 solid tissue samples= 132	Primary tumour samples and solid tissue normal samples	Microbiota profiling data at phylum, class, order, family, and genus levels followed by Linear discriminant analysis effect size (LEfSe) was used to determine the composition and abundance of the microbiota.	Not given	NA	Differences in critical oral microbiota between OSCC and normal controls. Genus *Treponema* showed higher abundance.	OSCC
3	Zhou et al. 2020, Cross-sectional study, China [16]	24 OSCC patients	Tissue sample	16 S rRNA gene sequencing	Not given	NA	The relative abundance of the following genera was increased in cancerous sites: *Treponema*,* Fusobacterium*,* Tannerella*,* Streptococcus*,* Parvimonas*,* Filifactor*, *Peptostreptococcus*, and *Carnobacterium*	OSCC
4	Hooper et al. 2007, Cross-sectional study, UK [17]	10	Tumour tissue	Fluorescence in situ hybridization (FISH)-using the universal eubacterial probe EUB338–FITC; 16 S rRNA gene sequences	Not given	NA	Bacteria detected in all sections of OSCC tissue. *Spirochetes*-specific primer, C90, detected no PCR products. *Clavibacter michiganensis* subsp. *tessellarius*,* Fusobacterium naviforme* and *Ralstonia insidiosa* were detected in ≥ 30% of the tumour specimens than non-tumour samples	OSCC
5	Nino et al. 2022, cross-sectional study, USA [18]	7 with OSCC (and 11 with colorectal Ca)	Tumour tissue	In situ spatial-profiling technologies and single-cell RNA sequencing.	Not given	NA	Integrated scRNA-seq revealed that the intratumoural microbiota is dominated by *Fusobacterium* (34%) and *Treponema* genus (29.8%), found more with tumour epithelial and monocyte-derived macrophage-v1 cell clusters.	OSCC and colorectal Ca
6	Singh et al. 2023, Cross -sectional study, India [19]	95 samples- 15 pre cancer and 60 OSCC; subdivided into early-stage (T1, T2) and late-stage (T3, T4), and 20 adjacent tumour tissue	Tissue from tumours	16 S rRNA sequencing; flow cytometry and immunohistochemistry analysis	Not given	NA	Precancer group –significant increase in *Rothia*,* Streptococcus*,* Arcanobacterium*,* Parvimonas*, and *Clostridiales* genus. Cancer group *-Capnocytophaga*, *Fusobacterium*, and *Treponema* genus Late cancer stages – *Capnocytophaga* Early cancer stage - *Fusobacterium*.	OSCC
7	Bebek *et al. 2012*, Case-control study, USA [20]	46 cases, 46 controls	*HNSCC* frozen tissue and paraffin-embedded samples of paired tumour and normal oral mucosa	Microbiomic profiling by 16 S rRNA sequencing	Not given	NA	Microbiomic profiling revealed seven different phyla represented in the oropharyngeal tissues which includes *spirochetes*.	HNSCC including oral cancer
8	Yang et al. 2022, Case control study, China [21]	27 cases and 15 controls	The surface of benign tumour tissues, OSCC tissues and identical tissues from healthy subjects were sampled using two identical sterile swabs.	Genomic DNA was extracted; 16 S rRNA gene sequencing of V3–V5 region	Not given	NA	The oral microbiota was composed mainly of *Proteobacteria* (31.76%, 35.00%), *Bacteroidetes* (30.13%, 25.13%) and *Firmicutes* (23.92%, 17.07%) in tumours and health, respectively. Tumour groups showed higher genera of *Treponema*,* Parvimonas*,* Staphylococcus*,* Micrococcus*,* Acinetobacter*,* Pseudomona*s, *Janthinobacterium*,* Loktanella*,* Propionibacterium*,* Catonella* and *Aggregatibacter* genera.	OSCC
9	Kylmä et al. 2023, Cross- sectional study, Finland [22]	201 **OPSCC** patients	Tissue	Immunohistochemically using tissue microarray	Not given	Td CTLP	Td-CTLP (dentilisin) detectable in tumour cells and associated with tumour stage, grade, regional metastasis, tumour site, smoking, and HPV status. Strong Td-CTLP (dentilisin) expressers had worse Disease Specific Survival (61%) than mild Td-CTLP (dentilisin) expression (79%).	Oropharyngeal Squamous cell carcinoma
10	Kylmä et al. 2020, Cohort study, Finland [23]	198 unselected consecutive OPSCC patients	Tissue	Immuno histochemical staining	Not given	Td-CTLP (dentilisin)	81% of the OPSCC samples showed Td-CTLP (dentilisin). Association between strong TLR 2- immunoexpression and poor DSS among the same HPV-negative subgroup of OSCC.	Oropharyngeal Squamous cell carcinoma
11	Kylma et al. 2022, Cohort study, Finland [24]	202 oropharyngeal squamous cell carcinoma **(OPSCC**) samples	Tissue from the tumours	Rgp, MMP-8, and MMP-9 immunoexpression was evaluated by immunohistochemistry; the immunohistochemistry of Td-CTLP (dentilisin)	Not given	NA	MMP-9 related to poor outcome in OPSCC, especially in HPV-negative disease, while Rgp immunoexpression in inflammatory cells associated with better disease-specific survival. No significant correlation between Td-CTLP and MMP9 in tumour cells.	Oropharyngeal Squamous cell carcinoma
12	Ye et al. 2021, Cross sectional study, China [25]	23 patients with Tongue Squamous Cell Carcinoma	Tissue	Next generation 16 S rDNA amplicon sequencing and functional prediction	*Treponema* might be involved in the pathogenesis of OSCC given its immunomodulatory and inflammatory capacity.	NA	At the phylum level, Bacteroidetes and Spirochetes were significantly increased in the cancer samples, while Actinobacteria decreased. At the genus level, Treponema_2 exhibited remarkable prevalence in the cancer samples.	OSCC
13	Yang et al. 2021, Cross sectional study, China [29]	65 samples form 23 patients	Tumour tissue, paracancerous tissue, saliva	16 S rRNA gene sequencing	Not given	NA	Spirochetes did not form core microbiome in OSCC but were present in 3.24% of all samples. In the early tumour stage (I/II), both tumour tissue and normal periodontal tissue had higher abundances of *Treponema* sp.	OSCC
14	Listyarifah et al. 2018, Cross sectional study, Finland [30]	60 patients – early-stage tongue SCC	Tissue	Tissue microarray and PCR	Not given	Td-CTLP (dentilisin)	High Td-CTLP (dentilisin) in patients ≤ 60 years old; 95% of tumour tissues were positive for Td-CTLP (dentilisin), which correlates with tumour invasion and size.	OSCC
15	Yan et al. 2023, Case control study, USA [31]	31 control and 35 cancer patients (*HNSCC*)	Tumour Tissue	Sequencing, PCR	Not given	NA	Significant increase in genus *Treponema* (*p* =.01) in the advanced-stage tumours. *Treponema* genus populations were rare in both control populations and early-stage tumours. *Treponema* genus was reported as unique to advanced‐stage tumours only. *Treponema* might be involved specifically in tumour progression rather than carcinogenesis,	HNSCC
16	Perera *et al. 2018*, Case control study, Australia [32]	27 males and 27 controls	Tissue	DNA Extraction and 16 S rRNA Sequencing	Not given	NA	*Treponema denticola* was not enriched in the OSCC tissues	OSCC
17	Su et al. 2021, Cross sectional study, Taiwan [33]	74	Oral swabs of surface of OSCC tumour lesions and contralateral tissue from normal healthy site	16 S rRNA gene amplicon sequencing and data processing	Not given	NA	*T. denticola*/spirochetes not part of the core microbiome in OSCC; *Treponema pectinovorum* was seen in lesion sites	OSCC
18	Gopinath et al. 2021, Case control study, Hong Kong, China [34]	48 cases; 46 controls	Subset − 44 matched surface swab and deeper tissues; 25 matched tissue and whole mouth fluid OSCC samples	16SrRNA sequencing	Not given specifically	NA	Genera *Treponema*,* Prevotella*,* Meiothermus*,* Sphingomonas* and *Mycoplasma* found abundant in deep tumour tissues	OSCC
19	Li *et al. 2020*, Cross ectional study, China [35]	Gingival SCC 10, periodontitis 15, healthy 15	Plaque, saliva and tissue	16SrDNA sequencing	Not given	NA	*T. denticola* found more in periodontitis; not prevalent at GSCC sites	OSCC
20	Rajakaruna et al. 2012, Cross sectional study, Japan [36]	66	Oral tissue sections and superficial and deep cervical lymph nodes	Real-time PCR	Not given	Td-CTLP (dentilisin), MSP	*T. denticola* not detected in any sample. *T. denticola* expresses MSP, which reduces neutrophil chemotaxis and phagocytosis.	Lymph nodes from head and neck cancer
21	Narayanan et al. 2023, Case control study, Sweden [37]	55 cases (periodontitis) and 44 controls (non-periodontitis); Orodigestive cancer, breast cancer, prostate cancer, gynaecological cancers, haematological malignancies, head and neck cancers and liver cancer	Subgingival plaque	DNA extraction, 16 S rRNA gene amplification, and sequencing	Not given	NA	Genera *Treponema*, *Fretibacterium*, and *Prevotella* abundant in periodontitis patients. The presence of periodontal inflammation correlated with *Prevotella*, *Treponema* and *Mycoplasma* genera. Cancer patients had abundance of phyla *Spirochetes*,* Fusobacteriota*,* Firmicutes and Proteobacteria*	Orodigestive cancer, breast cancer, prostate cancer, gynaecological cancers, haematological malignancies, head and neck cancers and liver cancer.
22	Soder et al. 2021, Cohort study, Sweden [38]	99 (only 2 cases of lip cancer)	Gingival crevicular fluid samples from periodontitis patients	Polymerase chain reaction (PCR)	Not given	NA	Three strong components observed: a) *Aa* b) Combination of *Tannerella forsythia* and *Treponema denticola* - not significantly associated with malignancy. c) *Porphyomonas gingivalis* and *Prevotella intermedia*.	Malignancy including oral cancer
23	Dahlstrom et al. 2024, Case control study, USA [39]	Thirteen HPV DNA-positive samples; 30 oral HPV DNA-negative samples from HPV-related **Oropharyngeal** and Uncommon Cancers Screening Trial of Men	Oral rinse sample	PCR was performed with 16 S rRNA primers	Not given	NA	HPV-positive: Increased abundance of phyla *Spirochaetota*,* Synergistota*, *Bacteroidota*, and decreased *Proteobacteria* *-* Genus *Treponema*,* Prevotella Fretibacterium*,* Kingella and F0058* more abundant HPV-negative samples: *Neisseria*, *Lactobacillus* abundant.	HPV associated cancers of the anogenital region and oropharynx
24	Shay et al. 2020, Case control study, USA [40]	46 cases of *HNSCC*, 46 non HNSCC controls	Oral wash samples	16 S rRNA and Internal Transcribed Spacer (ITS) gene sequencing on oral washes	Not given	NA	Eleven bacterial phyla were identified. Of these, *Firmicutes* (most dominant − 39.2%) followed by *Bacteroidetes (23.2%)*,* Actinobacteria (15.4%)*,* Proteobacteria (9.7%)* and *Fusobacteria* (8.0%) and *Spirochetes* (1.8%).	HNSCC
25	Sawant et al. 2023, Cross sectional study, India [41]	120 participants: healthy controls, long-term tobacco chewer, and oral squamous cell carcinoma patients	Salivary samples	16 S rRNA V6-V8 region amplification by PCR	Not given	NA	Potential biomarkers for tobacco chewers -Genera *Treponema*,* Tannerella*,* Campylobacter*,* Filifactor*,* Leptotrichia*,* Lautropia*,* Selenomonas*,* Cardiobacterium* Biomarkers for OSCC - *Capnocytophaga*,* Pseudomonas*,* Bifidobacterium*, *Peptostreptococcus*, *Paludibacter Mycoplasma*	OSCC
26	Pushalkar et al. 2011, Case control study, USA [42]	Three OSCC, two non-malignant controls	Salivary samples	454 DNA sequencing, of V4-V5 hypervariable region	Not given	NA	The phylum *Firmicutes* showed higher abundance in OSCC. *Spirochetes* (0.2%) were represented in both groups.	OSCC
27	Rai et al. 2020, Case control study. India [43]	OSCC patients – 25; Healthy − 24	Salivary samples	16 S rRNA gene sequencing of V3/V4 region; cytokine assay	Not given	NA	*Spirochetes* were identified, but with less abundance. Phylum *Bacteroidetes*,* Fusobacteria*,* Proteobacteria*,* Firmicutes* and *Actinobacteria* were abundant phyla in both control and OSCC samples.	OSCC
28	Mager et al. 2005, case control study, USA [44]	Non-matched group (45 OSCC + 229 control subjects); Age, gender, habit matched group (45 OSCC + 45 controls)	Unstimulated saliva samples	Checkerboard DNA-DNA hybridization	Not given	NA	OSCC – Significantly elevated counts of *P. melaninogenica*,* C. gingivalis*, and *S. mitis*. - Although *T. denticola* and *T. socranskii* were identified in the samples but not found significant.	OSCC
29	Börnigen et al. 2017, Case control study, USA [45]	121 OSCC patients; 242 controls	Oral rinse sample	Microbiome analysis; 16 S rRNA gene sequencing	Not given	NA	Periodontal microorganisms not detected (*Treponema denticola*,* Porphyromonas gingivalis*,* Tannerella forsythensis*,)	OSCC
30	Kang et al. 2009, case control study, Korea [47]	104 oncological (*head and neck cancer*, haematological neoplasia, solid tumours) patients and 52 healthy	Salivary samples	Microbial analysis using polymerase chain reaction	Not given	NA	*T. denticola* (ATCC 35405) was detected in 64.5% of patients with head and neck tumours and of these *T. denticola* (41.3%) was present in patients with haematological neoplasia and 71.1% in patients with solid tumours	*HNSCC*,* haematological neoplasia and other* tumours
31	Na et al. 2013, Cross sectional study, Korea [48]	36 subjects including 16 healthy volunteers, 11 periodontitis patients, and 9 OSCC patients	Gargling samples	PCR	Not given	NA	More in OSCC patients - *Streptococcus salivarius*, *Capnocytophaga ochracea and Gemella morbillorum*. Less in OSCC - *Prevotella nigrescens*, *Fusobacterium nucleatum*. Periodontitis patients- *Porphyromonas gingivalis*,* Treponema denticola*.	OSCC
32	Kaliamoorthy et al. 2021, Case control, India [28]	30 OSCC, 30 non cancerous	Tissue	PCR	Not given	NA	*Treponema denticola* detected in 8/30 OSCC samples and 26.6% of cases diagnosed with OSCC	OSCC
33	Kylma et al. 2018, Cohort study, Finland [49]	201 **oropharyngeal cancer**	Tissue from the tumours	Immunohistochemistry	Td-CTLP (dentilisin) helps *Treponema denticola* in epithelial invasion by degrading basement membrane	Chymotrypsin-like protease (Td-CTLP) (dentilisin)	Td-CTLP (dentilisin) found in tumours and associated with HPV status. Td-CTLP (dentilisin) expression indicates poor 5-year Disease Specific Survival	OPSCC
34	Chen et al. 2021, cross-sectional study, Taiwan [46]	34 OSCC-OSF; 18 OSF	Salivary samples	16 S rRNA gene sequencing	Not given	NA	*Spirochetes* (1.6% ± 2.6%) were detected in 52 samples; spirochetes did not form the core of the OSCC group. OSCC-OSF group had significantly more *Treponema* sp. HMT-270. *Treponema* sp. HMT-927 was part of the OSF signature species but was also detected by artificial intelligence in the OSCC-OSF group.	OSCC
35	Shin et al. 2017, Case control study, USA [26]	72 tissue samples from 34 *HNSCC* subjects	Tissue samples (normal, primary, metastatic)	RNA Extraction and cDNA Synthesis, Microbiome Sequencing and Analysis.	*Treponema* species contribute to tumour progress through protease induction which can destroy basement membranes.	NA	Primary HNSCC tissues- increased abundance in phyla *Bacteroidetes*,* Proteobacteria*,* Spirochetes* and *Fusobacteria* *Treponema* genus were increased in oral cavity HNSCC samples.	HNSCC

### Type of sample

Tumour tissue has been used as the sample by Cai et al. [14], Li et al. [15]., Zhou et al. [16]., Hooper et al. [17], Nino et al. [18], Singh et al. [19]., Bebek et al. [20]., Yang et al. [21], Kylma and others [22–24], Ye et al. [25]., Shin et al. [26]., Kaliamoorthy [27, 28] Yang et al. [29]., Listyarifah et al. [30]., Yan et al. [31]. and Perera et al. [32]. Su and others studied oral swabs [33], while Gopinath utilized swabs from the tumour surface and from deep within the tumour tissue [34]. Li et al. [35] obtained plaque, saliva and tissue samples. Rajakaruna et al. assessed the gingiva, oral mucosa and lymph nodes [36]. Narayanan et al. [37]. used subgingival plaque samples while Soder et al. evaluated gingival crevicular fluid [38]. Oral rinse and salivary samples were featured in studies by Dahlstrom et al. [39], Shay et al. [40]., Sawant et al. [41], Pushalkar et al. [42], Rai et al. [43], Mager et al. [44], Bornigen et al. [45], Chen et al. [46], Kang et al. [47], Na et al. [48] and other researchers.

### Method of analysis

Sequencing of the bacterial 16 S rRNA gene [14, 16, 19–21, 25, 26, 29, 32–37, 40, 41, 43, 45] has been used widely for assessing the oral microbial profile after DNA extraction. Li et al. [15] used linear discriminant analysis of effect size (LEfSe) to determine bacterial composition and abundance. Other techniques used for the identification of the microbiota include polymerase chain reaction (PCR) [28, 30, 31, 38, 42, 47, 48], fluorescence in situ hybridization (FISH) [17], single-cell RNA-sequencing method (invasion–adhesion-directed expression sequencing) [18], checkerboard DNA-DNA hybridization [44] and immunohistochemistry [22–24, 49].

### Taxonomy

The genus ‘*Treponema*’ was identified in several studies [14, 16, 18, 19, 21, 34, 39, 41], but not as a species, which could have been a limitation of the diagnostic technique. Bacteria phylogenetically similar to *T. denticola* have been identified in some studies [14, 33], but not *T. denticola*. Su et al. do not suggest *T. denticola/spirochetes* as part of the core microbiome in OSCC but mention *Treponema pectinovorum* at lesion sites [33]. Chen et al. [46]. suggest a subspecies of *Treponema*,* Treponema* sp. *HMT-270* is relatively abundant in those with OSCC and oral submucous fibrosis (OSCC-OSC group) compared to OSF alone. The study by Cai et al. mentions *T. medium* but focuses primarily on the role of *Fusobacterium nucleatum* [14].

The phylum ‘*Spirochetes*’ has been found in the oral samples of several studies [20, 37, 40, 42, 43]. While some opine *T. denticola* is not associated with OSCC [32, 35, 36, 38, 44, 45, 48], others state an association [26, 28, 31, 47].

### Detection of virulence factors

Specific attempts to isolate spirochetes in the OSCC samples have sometimes failed [17]. Studies, therefore, have indirectly evaluated its effects through its chymotrypsin-like protease [Td-CTLP (dentilisin)] to delineate its association with OSCC [24, 30, 49]. They found this virulence factor in high quantities in oro-pharyngeal carcinomas [23, 49]. The ability of Td-CTLP (dentilisin) to degrade basement membrane components is another likely mechanism in oral carcinogenesis [26, 49]. Td-CTLP (dentilisin) seemed to significantly associate with site, stage and grade of tumours, metastasis. Furthermore, it was also found with the habit of smoking and poor Disease-Specific Survival (61%) [22]. Listyarifah [30] noted that Td-CTLP (dentilisin) was highly immunopositive in older patients, which correlated with tumour size and depth of invasion, suggesting a relapse.

### Presence of other microorganisms

Other microorganisms also coexist in the oral biofilm. Sawant et al. [41] suggested potential biomarkers for tobacco chewing like *Treponema*,* Tannerella*,* Campylobacter*, *Filifactor*,* Leptotrichia*,* Selenomonas*,* Lautropia*, and *Cardiobacterium*, while *Capnocytophaga*,* Peptostreptococcus*, *Pseudomonas*,* Paludibacter*,* Bifidobacterium*, and *Mycoplasma* were likely biomarkers for OSCC [41]. Soder et al. [38] suggested *Aggregatibacter actinomycetemcomitans* showed a stronger tendency to be associated with various malignancies than *T. denticola* and *T. forsythia* [38]. Only about 0.2% spirochete*s* were observed in OSCC compared to *Fusobacteria* (5%), *Proteobacteria* (10%) and *Actinobacteria* (14%) with sparse amounts of SR1 (0.6%) and uncultured species like TM7 (0.2%) [42]. Mager et al. [44] found *T. denticola* and *T. socranskii* to be present in the OSCC samples but higher salivary counts of *C. gingivalis*,* P. melaninogenica* and *S. mitis* were suggested as diagnostic indicators of OSCC. The genus *Dialister* was more abundant in oral rinse samples of oral and oro-pharyngeal cancer [44, 45] In contrast, Nino et al. found *Fusobacterium* (34%) and *Treponema* (29.8%) to be the dominant genera within tumour tissues, mostly seen with the clusters of epithelial and monocyte-derived macrophage-v1 cells [18]. Similarly, studies demonstrate higher proportions of the genera *Capnocytophaga*,* Fusobacterium*,* Treponema* and other species in cancer groups [16, 19, 21, 25, 26].

### Tumour tissue penetration of spirochetes

Approximately 54% of the clinical studies found spirochetes or *Trep*o*nema* (genus/species) or its virulence factor abundant at cancer sites. *T. denticola* was detected in 64.5% of patients with head and neck tumours [47]. Yang et al. [29] observed both tumour-tissue and normal periodontal tissue showing higher abundances of *Treponema sp.* The genus *Treponema* was proposed to be unique to advanced-stage tumours [31] by some authors, whereas others delegate them to early cancers. In one of the included studies, the *Treponema* genus was abundant in deep tumour samples along with other genera like *Prevotella*,* Sphingomonas*,* Meiothermus* and *Mycoplasma* [34]. Kaliamoorthy et al. [28] detected *T. denticola* in 26.6% of cases with OSCC. Rajakaruna et al. [36] could not identify *T. denticola* in tumour samples. In contradiction, the studies by Shay et al. [40] and Bebek et al. [20] have located spirochete*s* within tumour tissues. Rai et al. [43] noted that although *spirochetes* were present, they were not abundant.

### Proposed mechanisms for oral carcinoma pathogenesis

The immunomodulatory and inflammatory activity of the *Treponema* genus, as stated in the article by Ye et al., lends credence to its role in OSCC pathogenesis [25]. The cross communication between two signalling pathways, TLR/MyD88 and integrin/FAK, is another proposed mechanism [14]. With no specific reference to the genus *Treponema*, Gopinath et al. [34] hypothesise that when the homeostasis of the nitrate reduction pathway is disturbed, the microbiota may produce ammonia and nitrites, which can facilitate the proliferation of cancer cells.

An overview of clinical studies reveals diverse findings regarding the presence of the *Treponema* genus and its relation to OSCC. Ten studies detected the *Treponema* genus in tumour tissues [14, 16, 18, 19, 21, 31, 33, 34, 39, 41], with one proposing a role in cancer progression [31]. Two studies linked *Treponema denticola* to an increased cancer risk in patients with predisposing factors such as *Human Papillomavirus* (HPV) infection or tobacco use [23, 49]. *T. denticola* was suggested as a significant diagnostic marker for malignancy in two studies [28, 47]. Seven studies did not favour *T. denticola* in OSCC [32, 35, 36, 38, 44, 45, 48]. One study found *T. denticola* in periodontitis patients rather than those with oral squamous cell carcinoma (OSCC) [29]. Four studies observed the virulence factor Td-CTLP (dentilisin) in tumours and associated tissues [22, 24, 30, 49]. Additionally, *spirochetes* were observed in OSCC samples in five studies [20, 37, 40, 42, 43], though four of them reported their presence in low abundance [20, 40, 42, 43].

#### Reviews

A ‘title and abstract’ search facilitated the selection of 21 review papers on the topic from the period 2019 to 2024. This scoping review comprises mainly narrative reviews and one systematic review and meta-analysis [50]. Eight papers were from the USA, two from Romania, one from Iran, five from India, three from China and one from Colombia and Poland (Table 2).

**Table 2:** Summary of review articles

S. No	Author	Inference of the study	Associated cancer type
1	Kaliamoorthy et al. 2024, India [27]	Direct and indirect mechanisms by which *T. denticola* induces OSCC	OSCC
2	Xiao et al. 2020, China [50]	*Treponema denticola* not associated with increased cancer risk.	Role in carcinogenesis in general
3	Gonzalez et al. 2021, USA [51]	Chronic inflammation and persistent Gram-negative infection seen in periodontal disease settings is also associated with head and neck cancers	HNSCC
4	Constantin et al. 2023, Romania [54]	Genus *Treponema* was observed commonly in head and neck cancers. The genus *Treponema* was described to show significant abundance changes during radiation therapy and negative interaction with probiotics. Its presence along with other Gram-negative species such as *Fusobacterium*,* Prevotella*,* Porphyromonas etc.*. is linked to the chronic inflammation in tumours.	Head and neck carcinoma
5	Beltran et al. 2021, Columbia [55]	Tobacco use increases presence of *Treponema spp. T. denticola* was seen with intra-tumour OSCC tissue in one of thirteen studies reviewed.	OSCC
6	Sukmana et al. 2024, Iran [56]	*T. denticola* protease, dentilisin, is crucial to tumour invasiveness. It is also linked to early carcinoma in younger individuals with poor prognosis.	OSCC
7	Chattopadhyay et al. 2019, India [57]	*Treponema* genus was more abundant in swabs of patients with OSCC.	OSCC
8	Gheorghe et al. 2022, Romania [58]	*T. denticola* is linked to OSCC.	OSCC
9	Fitzsimonds et al. 2020, USA [59]	*T. denticola* is less abundant in health and has high proteolytic action.	Oral carcinoma
10	Teles et al. 2020, USA [60]	Periodontal pathogens such as genera *Fusobacterium*, *Treponema*,* Prevotella*, *Parvimonas*,* Campylobacter* and *Filifactor*, abound in oral carcinomas.	Oral carcinoma
11	Acharya et al. 2024, India [61]	*T. denticola* has virulence factors such as dentilisin for adherence, immunomodulation and cytotoxicity; flagella for tissue penetration and chemotaxis; and hemin binding protein.	OSCC
12	Lamont et al. 2023, USA [64]	*P. gingivalis*, *T. denticola* and *F. nucleatum* enhance tumour aggression via a cross communication between integrin/FAK and TLR/MyDD88 pathways. Further, viable *T. denticola* was seen within gingival epithelium.	Role in carcinogenesis in general
13	Peng et al. 2022, China [65]	*T. denticola* regulates cell cycle through TGF-β pathway activation, inhibits cell apoptosis, and promotes OSCC proliferation.	OSCC
14	Sedghi et al. 2021, USA [66]	*T. denticola* affects the cell barrier function directly. Dentilisin activates TLR2, which induces proinflammatory action of MMPs. LPS from *T. denticola* promoted cell migration. Mutual interaction between *T. denticola* and *P. gingivalis*.	OSCC
15	Diwan et al. 2023, India [67]	Nisin reverses the effects of *T. denticola* in aggressivity of OSCC via TLR/MyD88 mediated Integrin alpha5/FAK signalling	OSCC
16	Silbergleit et al. 2020, USA [68]	*T. denticola* lacks LPS. Dentilisin effects include loss of epithelial cell contacts, rapidly penetrates the cell layers; increases epithelial permeability, degrades basement membrane, degrades tight junctions, degrades TNF-alpha, has ability to induce MMP-2, linked to immune evasion and tumour invasion. It also activates cell proliferation signals (ERK1, ERK2), and mediates apoptosis through JNK and p38.	Role in carcinogenesis in general
17	Stasiewicz & Karpinski, 2022, Poland [69]	*T. denticola* increases inflammatory cytokine expression (IL-1beta, TNF-alpha, IL-6) and MMP-9. MMPs important in tumour invasiveness and metastasis.	Role in carcinogenesis in general
18	Radaic et al. 2021, USA [70]	*T. denticola* enhances OSCC migration, invasion and tumoursphere formation and these were abrogated by the antimicrobial bacteriocin. *T. denticola* can degrade zonula occludens-1, claudin-1, and occludin − 1. *T. d*enticola has been linked to epigenetic modulation in vitro.	Role in carcinogenesis in general
19	Irfan et al. 2020, USA [62]	OSCC risk was found associated with *HPV*,* Porphyromonas gingivalis* and *Fusobacterium nucleatum. Treponema denticola* was not specifically discussed.	OSCC
20	Ananthalakshmi et al. 2021, India [63]	*T. denticola* among other bacteria that are important in the carcinogenesis process.	Oral carcinoma
21	Zhang et al. 2019, China [84]	*T. denticola* has virulence factors like major surface protein complex, CTLP (dentilisin) and periplasmic flagella. *T. denticola* modulates inflammation by suppressing antimicrobial peptide human β-Defensin-2 and IL-8. OSCC patients have a high incidence of *T. denticola*.	Oral carcinoma

### Periodontal infection and oral carcinoma

Gonzalez & Watts [51] hinted periodontal infection with *T. denticola* is associated with the occurrence of OSCCs. Several reports indicate a 2 to 5-fold increased risk for OSCC in those with periodontitis [52]. However, one study found no such association [53]. Constantin [54] and Beltran et al. [55] made similar observations in oral tumour tissues.

### Presence of T. denticola at OSCC sites

A systematic review by Xiao et al. [50] found *T. denticola* did not have a propensity for increased cancer incidence in 39 studies comprising 7184 participants [50]. Beltran et al. [55] found *T. denticola* to be present within tumours in only one of thirteen studies. However, the search considered many types of cancers, not restricted to oral carcinomas. They mentioned that very few studies focus on *T. denticola*, with emphasis on other microorganisms such as *F. nucleatum* and *P. gingivalis*.

Sukmana et al. [56] noted *T. denticola* in eight oral squamous cell carcinoma tissue specimens, but none of the control tissue specimens. Interestingly, it was present in the early stages of tongue carcinoma. The presence of ‘dentilisin’ was found to suggest a relatively poorer prognosis. *T. denticola* was abundant in swabs of patients with OSCC [57, 58]. Similarly, Fitzsimonds et al. [59] found *T. denticola* to be less abundant in healthy states.

Teles et al. [60]. found T. *denticola* to be more abundant in oral carcinoma than in non-cancer populations. Acharya et al. [61] observed *T. denticola* in OSCC along with the virulence factors, flagella and hemin-binding protein.

### Presence of other microorganisms at OSCC sites

Species other than *T. denticola* were associated with a higher risk for OSCC, like *Prevotella*,* Fusobacterium*,* Parvimonas*,* Bacteroidetes*,* Leptotrichia*,* Selenomonas*,* Capnocytophaga*,* Pseudoalteromonas*,* Solobacterium*,* Alloprevotella*,* Peptostreptococcus*,* Clostridium* and *Porphyromonas gingivalis* [54]. Teles et al. [60]. highlighted the genera *Fusobacterium*,* Prevotella*, *Campylobacter*,* Parvimonas* and *Filifactor* were relatively abundant in oral carcinomas. Irfan et al. [62] mention *HPV*,* Porphyromonas gingivalis*,* Fusobacterium nucleatum* and different bacterial genera and taxa in oral carcinoma samples. Ananthalakshmi et al. [63]. underscored *Streptococcus anginosus* as a potential diagnostic marker. They also reported salivary elevations in species such as *Prevotella melaninogenica*,* Capnocytophaga gingivalis* and *Streptococcus mitis.* Furthermore, the keratinising forms of oral carcinoma possessed higher numbers of *Streptococcus*,* Prevotella*,* Veillonella*,* Fusobacterium*,* Porphyromonas*,* Haemophilus*,* Clostridium*,* Enterobacteriaceae* and *Actinomyces.*

### Proposed mechanism

Kaliamoorthy [27] described various mechanisms by which *T. denticola* can induce carcinogenesis, directly and indirectly, including tumour promotion, cell migration, depth of invasion, tumoursphere modification, enhancement of inflammatory mediators, and macrophage alteration to the M2 type. Persistent infection by *T. denticola* triggers the reactive oxygen species, which can damage the DNA of proliferating cells [27]. Fitzsimonds et al. [59] suggested that *T. denticola* has high proteolytic activity attributed to dentilisin, which favours epithelial cell invasion.

Lamont et al. [64] suggested that *T. denticola* resides within epithelial cells for extended periods by avoiding lysosomal degradation. It evades chemotaxis by neutrophils and activation of the immune system. It can also regulate IL-36, a proinflammatory cytokine.

 In vitro studies on *T. denticola* involvement in OSCC initiation and progression [65, 66] also hinted at *T. denticola*’s ability to disrupt cell barrier function, enhance cell migration via its lipopolysaccharide and induce proinflammatory effects via Toll-like receptor 2 and matrix metalloproteases. Diwan et al. [67] discussed the role of anti-cancer agents, probiotics and Nisin, in reversing the effects of *T. denticola’s* aggressivity in cancer.

Silbergleit et al. infer that through dentilisin, *T. denticola* can disrupt epithelial cell contacts, penetrate the cell layers, increase epithelial permeability, degrade TNF-alpha, and induce MMP-2, thereby evading immune responses and allowing tumour invasion [68]. They also suggested that it can activate signals mediating cell proliferation and apoptosis [68]. *T. denticola* can increase the level of proinflammatory cytokines and MMP-9, essential for tumour invasiveness and metastasis [69]. Radaic et al. [70] attribute *T. denticola* to genetic and epigenetic changes during cell division.

In summary, the review papers discussed pathogenic mechanisms based on in vitro studies. Persistent Gram-negative infections and chronic inflammation, such as those found in periodontitis, were believed to modulate the tumour environment by activating the TGF-β pathway and inhibiting cell apoptosis, affecting the cell cycle [65, 68]. *T. denticola* appeared to influence cellular division, chromatid segregation, and histone methylation and acetylation.

It is important to recognise that *Treponema denticola* rarely acts in isolation but frequently co-occurs with other major periodontal pathogens such as *Porphyromonas gingivalis* and *Fusobacterium nucleatum*. Synergistic interactions among these species—including co-aggregation, cooperative hemin acquisition, and outer membrane vesicle (OMV) cross-talk—likely amplify their pathogenic potential and may underlie the observed effects in both experimental and clinical settings.

Moreover, even in contexts where *T. denticola* abundance is relatively low, its functional activity remains biologically relevant. The organism’s high protease burden, particularly dentilisin, together with the release of OMVs enriched in virulence factors, can exert significant host-modulatory effects that outweigh its numerical representation within the microbial community. These considerations caution against attributing causality to *T. denticola* alone and instead support viewing its contribution within the framework of polymicrobial synergy and functional activity.

#### Database studies

We also evaluated observational studies from three databases (2022-23) (Table 3). Hamada et al. [71]. included 154 patients from The Cancer Genome Atlas (TCGA) and The Cancer Microbiome Atlas datasets comparing normal tissue and tumours for microbiome analysis. They identified *Treponema* as genus only in the TCMA database.

**Table 3:** Summary of database studies

S. No.	Author	Sample type/Method of detection of T. denticola	Inference of the study	Associated cancer type
1	Kim et al. 2022, Korea [72]	Kraken-TCGA (The Cancer Genome Atlas) -Raw-Data (*n* = 17,625); Primary tumours were selected from *HNSCC;* Site of occurrence as either oral cancer (alveolar ridge, buccal mucosa, floor of the mouth, hard palate, lip, oral cavity, and oral tongue) or non-oral cancer (base of tongue, hypopharyngeal, larynx, oropharynx, and tonsil) Linear discriminant analysis effect size method	The microbiome found enriched in cancers of the oral cavity were *Treponema*,* Fusobacterium*,* Leptotrichia*,* Selenomonas. At* non-oral sites - *Clostridium* and *Pseudoalteromonas*.	Head and Neck Cancer
2	Sharma et al. 2022, India [73]	Sample datasets (cancer patients and tobacco chewers)/ 16 S rRNA	Negligible in OSCC compared to 0.6% *Treponema* genus in tobacco chewers; not part of the core microbiome in OSCC	OSCC
3	Hamada et al. 2023, Japan [71]	Normal tissue (*n* = 22) and primary tumours of *HNSCC* patients (*n* = 154)/ Tissue microbiome profiling	Microbe genera identified: *Treponema*,* Porphyromonas*,* Actinomyces*,* Prevotella*,* Alloprevotella*,* Aggregatibacter*,* Campylobacter*,* Capnocytophaga*,* Fusobacterium*,* Streptococcus*,* Rothia*,* Haemophilus*,* Neisseria*,* Veillonella*,,* Granulicatella*,* Lactobacillus*,* Leptotrichia and Mycoplasma.* *Treponema* not associated with survival rates.	Head and neck cancer

Kim et al. [72]. evaluated datasets from Kraken-TCGA comprising 691 samples of head and neck cancer, subdivided into 172 DNA Whole-genome sequencing data and 519 RNA sequencing data. Comparison of tumours with clinical data sets showed genus *Treponema* is enriched in the microbiome of head and neck carcinomas but not in non-oral carcinomas. Microbiome data derived from The Cancer Genome Atlas (TCGA) requires careful interpretation due to the inherent risk of contamination and methodological variability. Bacterial reads obtained from RNA-seq and whole genome sequencing (WGS) platforms may differ in both sensitivity and bias, with RNA-seq favouring transcriptionally active taxa and WGS providing broader but potentially noisier coverage. Moreover, low microbial biomass in tumour samples increases susceptibility to spurious signals from kit contaminants, reagent-derived DNA, and batch effects. These limitations must be acknowledged when interpreting associations between bacterial taxa such as *Treponema denticola* in OSCC.

A database study [73] on tobacco chewers and cancer patients with 16 S rRNA sequencing found *Treponema* genus in OSCC lesions, albeit in negligible quantities compared to tobacco chewers (0.6%). It was not a part of the core microbiome in OSCC.

#### Preclinical animal model studies

Table 4.

**Table 4:** Summary of animal studies

S. No.	Author	Sample type/Method of detection	Inference of the study	Associated cancer type
1	Kamarajan et al. 2020, USA [74]	Human OSCC cell lines, UM-SCC-14 A (floor of mouth) and HSC-3 (tongue) *T. denticola* (ATCC 35405)/ Scratch migration, tumoursphere and matrigel invasion assay	By triggering TLR/MyD88 and integrin/FAK cross communication, *T. denticola* mediated an aggressive form of oral carcinoma. *T. denticola* increased cell migration and cell invasion. But the proliferative and apoptotic effects were less.	OSCC
2	Peng et al. 2022, China [75]	Murine tumours/ Tumourigenesis study, immuno- histochemical analysis	Chronic infection of mice with *T. denticola* enhanced tumour growth. Tumour cells showed upregulation of Ki67 indicating strong proliferative activity.	OSCC

An animal model study by Kamarajan et al. [74]. used scratch migration assay, matrigel invasion assay and tumour sphere assay to assess the effect of microbiota on OSCC cell lines. *T. denticola* upregulated expression of integrin alpha V significantly. Lipopolysaccharides derived from *T. denticola* promoted OSCC cell migration. *T. denticola* presence mediates aggressivity in cancer lesions triggered by a cross-communication between integrin/FAK and TLR/MyD88, enhancing cell migration. Hence, *T. denticola* has indirect effects through Toll-like receptor mediation [74]. They opined that *T. denticola* enhances cell migration and invasion rather than cancer-initiation. *T. denticola* lipopolysaccharide components were distinct from other lipopolysaccharides as lacking heptose, 3-deoxy-d-manno-2-octulosonic acid, and β-hydroxy fatty acids [74], which explains its effects on cells. Nisin inhibition of this pathogen mediator crosstalk provided proof of this concept. Nevertheless, since a polymicrobial inoculum in which *T. denticola* was co-inoculated with other key periodontal pathogens, *Fusobacterium nucleatum* and *Porphyromonas gingivalis*, was used in the study, the outcome may be due to a synergistic periodontal pathogen consortium that more accurately reflects the complex biofilm ecology of the oral cavity.

Tumour weight and volume in live *T. denticola* groups were significantly greater than in the control groups with heat-killed *T. denticola* (0.256 g vs. 0.077 g; 126.622mm^3^ vs. 50.377mm^3^), suggesting it has potential for increasing tumour size [75].

Experimental outcomes can also vary depending on whether live or heat-killed *T. denticola* cells were used. Findings indicate that many host responses are viability-dependent, suggesting an active role for bacterial metabolism and its secreted factors. At the same time, studies employing isolated virulence components such as lipopolysaccharide or outer membrane vesicles demonstrate that some phenotypes can be reproduced independent of bacterial viability, underscoring the contribution of discrete structural and secreted molecules.

#### In vitro studies

Table 5.

**Table 5:** Summary of in vitro research

S. No.	Author	Sample type/Method of detection	MOA of T. denticola in OSCC pathogenesis	Virulence factor studied	Inference of the study	Associated cancer type
1	Mahtout et al. 2011, Canada [77]	Immortalized human oral epithelial cell line GMSM-K/ Real-time PCR, Immunofluorescence analysis, ELISA	Lipopolysaccharide of *Treponema denticola* (ATCC 35405) increased CD55 and CD59, CD46 after 48 h.	*Treponema denticola* lipopolysaccharide	*T. denticola* lipopolysaccharide can control CRP expression in oral epithelium.	Oral cancer
2	Nieminen et al. 2018, Finland [76]	Orodigestive tumour tissues (Oral, tonsillar, and oesophageal squamous cell carcinomas); Gingival tissue from periodontitis patients -positive controls/ Immunohistochemistry	*Treponema denticola*-CTLP (dentilisin) activates MMP-8, 9	Td-CTLP (dentilisin)	*Treponema denticola* chymotrypsin-like proteinase was present in most studied tumour tissues. CTLP (dentilisin) converts MMPs into active forms, degrades TIMPs and inhibits complement, thus showing immunomodulatory activity, thereby promoting carcinogenesis.	Orodigestive carcinomas
3	Jones et al. 2019, USA [78]	*T. denticola* (strain 35405, 33520, OTK) culture, murine neutrophils/ Enzyme linked immunosorbent assay, qRT PCR, immunoprecipitation	-	Msp	Msp manipulates neutrophil chemotaxis by signalling it. It impairs the immune response, which can contribute to chronic inflammation that is a risk factor for cancer development	Role in general carcinogenesis
4	Peng et al. 2022, China [75]	OSCC cell line Cal-27 (ATCC CRL-1628)/ Cell proliferation assay, tumourigenesis assay, Transmission electron microscopy, flow cytometry, sequencing, PCR	*T. denticola* (ATCC 35405) could trigger TGF-β1, -β2, and -β3 mRNA expressions, affecting cell cycle regulation and cell proliferation. It also increased TLR4 and NF-kB expression.	-	*T. denticola* invasion regulated the cell cycle by allowing OSCC cell proliferation and inhibiting apoptosis. The *spirochete* enhanced TGF-β expression by activating TLR4-MyD88–NF-kB signaling.	OSCC

The 4 in vitro studies appraised in this scoping review [76] used immunohistochemistry [76], Enzyme linked Immunosorbent Assay, cell proliferation assay, tumourigenesis assay, Transmission Electron Microscopy, and flow cytometry [75].

Peng et al. [75]. observed that at appropriate concentrations, *T. denticola* directly enhances OSCC cell proliferation through the TGF-β pathway. Further, it can also modulate the cell cycle inhibiting apoptosis. Td-CTLP (dentilisin) activates matrix metalloproteases 8 and 9 in most tumours. It inhibited complement factor C1q and caused degradation of tissue inhibitors of metalloproteases, TIMPs 1, 2 and α−1-antichymotrypsin [76]. This immunomodulation is suggestive of its cancer-promoting ability. *T. denticola* lipopolysaccharide increased CD46, CD55, and CD59 protein expression, and induced upregulation of epithelial C-reactive protein (CRP) [77]. Tumour cells also showed increased expression of CRPs. In another study, Jones et al. [78] demonstrated that *T. denticola’s* Msp protein released from its outer membrane vesicles has a role in suppressing neutrophil chemotactic response. This feature promotes bacterial survival and dysbiosis of the biofilm [78].

Letter to the editor.

A letter to the editor concerning the oral microbiome in early oral cancer [79] speculates that *Treponema lecithinolyticum* found at sites with periodontitis drives oral tumour progression with no specific explanation.

### Microbial profiling and other characteristics of the included studies

The clinical studies included in the review have adequate sample sizes to assess the microbial profile. The confounders mentioned in the studies have either been controlled or considered as variables. The studies show different methods of microbial profiling. Genus-level identification, common in 16 S rRNA sequencing, offers a broad overview of microbial communities but may mask critical differences between species, especially when some are pathogenic and others are not. Species-level profiling is clinically relevant as it precisely identifies beneficial vs. harmful organisms. However, whole-genome sequencing (WGS), metagenomic and shotgun sequencing techniques, and species-specific PCR or FISH probes are required but are costlier, more complex, and computationally demanding. Further, closely related species can be difficult to distinguish genetically. While genus-level data are beneficial for ecological trends, species-level identification is essential for accurate diagnosis, understanding disease mechanisms, and guiding targeted interventions. Single-cell RNA sequencing offers high-resolution insights into host-microbe interactions and gene expression. However, it is complex and costly. The checkerboard DNA–DNA hybridisation simultaneously detects multiple known species across many samples, but it cannot detect unknown microbes and is only semi-quantitative. Immunohistochemistry (IHC) detects microbial antigens and host markers in tissues, providing spatial and immune context. However, it depends on antibody specificity and lacks broader microbial coverage. Each method offers valuable insights. Combining the different techniques is often necessary for assessing the comprehensive microbe-host interaction in the oral environment. However, the variability in the methods used in each of the studies included in the review may influence the reliability of the studies.

## Discussion

Multiple factors contribute to OSCC aetiology within the background of genetics and the epigenetic milieu. Acid reflux, poor dental hygiene, consuming smoked or fried meat, candidal and viral infections are conducive to changes in the oral habitat, enabling dysbiosis [54]. Antibiotic resistance and treatments such as radiotherapy or chemotherapy may induce a dysbiotic environment. We have excluded such studies from the search data of the present review. Several studies included individuals exposed to tobacco.

A study found augmented tumour size and numbers in mice models inoculated with the oral microbiome compared to germ-free control rodents [80] subjected to an oral carcinogen (4-NQO), attributed to elevated inflammation in the tumour microenvironment. However, it is not clear whether the microbiome triggered the tumour or whether other factors induced changes in the microbiome. The authors surmise that microbial signals modulate cancer by influencing genetic instability or through mutation, not merely through the route of inflammation. Differences may also exist between the murine and human microbiome [80].

Studies have emphasised species abundance rather than qualitative changes. It is not clear whether the depletion of commensal flora could herald oncogenesis. *Firmicutes* and *Actinobacteria* phyla decrease with a concomitant increase in *Fusobacterium nucleatum* [54].

In this regard, *Porphyromonas gingivalis* and *Fusobacterium nucleatum* species have often been implicated in OSCC [3, 81]. However, *Treponema denticola*, which co-exists with *P. gingivalis*, is scarcely deliberated and thus forms the focal point of this scoping review.

*Spirochetes* form one of the six main phyla in healthy individuals, collectively contributing 96% of the taxa in the oral cavity [82]. The reference to *T. denticola* in studies on OSCC originates from its presence in periodontal disease, a proposed risk factor for oral carcinomas [67].

*T. denticola* is a motile spirochete found in the red complex group of periodontal pathogens, concomitant with *Porphyromonas gingivalis* and *Tannerella forsythia.* Theories proposed to explain its role in oral carcinogenesis are chronic inflammation, production of virulence factors, immune evasion, and synergy with other oral bacteria.

### Mechanism of action

**Fig. 2:**
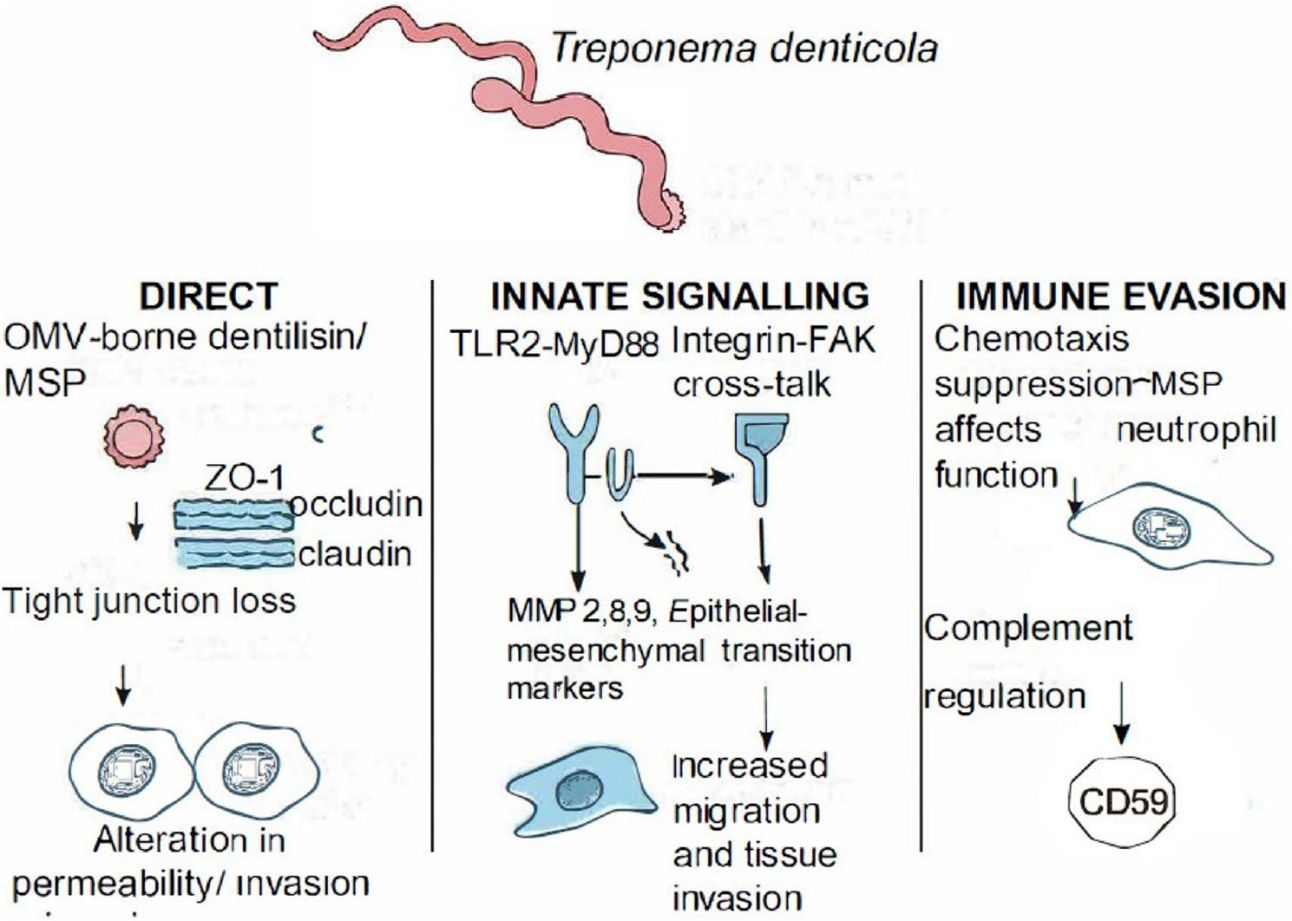
Mechanisms of action of *T. denticola*

*T. denticola* may play a role in prolonging inflammation in the oral environment by continuously releasing inflammatory mediators such as prostaglandin E2 (PGE2), interleukin-6 (IL-6) and tumour necrosis factor-alpha (TNF-α). These molecules induce cell proliferation and promote angiogenesis [83]. Dentilisin can degrade cytokines (including TNF-α/IL-8; hBD2) and modulate TLR2 signalling [84]. *T. denticola* can also inhibit the migration of neutrophils and fibroblasts [85]. *T. denticola* facilitates extracellular matrix degradation by producing metalloproteases 8 and 9. This degradation not only promotes tissue destruction but also facilitates the invasion of cancer cells into surrounding tissues [86]. *T. denticola* lipopolysaccharide exposure upregulates complement regulators (e.g., CD59), aiding immune evasion [77]. (Fig. 2)

### Virulence factors

Several articles highlight the virulence of *T. denticola* through dentilisin, a cell membrane-associated protease complex that can cleave alanyl bonds [87]. It facilitates bacterial coaggregation, complement activation, and immune evasion and inhibits the haemostasis system. Dentilisin activates host-expressed TLR2 receptors [88] and affects TLR 7 and 9 expressions [30]. Immune evasion and cytotoxicity occur by membrane blebbing, vacuolization, and the release of cytosolic enzymes. Further, dentilisin can induce the production of damaging superoxide radicals from neutrophils. It can degrade cytokines such as IL-8 and TNF-alpha.

Dentilisin activates matrix metalloproteases 2, 8 and 9 but resists the effects of tissue inhibitors of matrix metalloproteases, TIMPs 1 and 2. *T. denticola* can invade and survive within cells, dependent on dentilisin activity by resisting endolysosomal degradation. Dentilisin was evident in the cytoplasm of approximately 87% of oropharyngeal carcinoma tissues. Dentilisin’s effects in chemotaxis, adherence, promoting the loss of epithelial cell contacts by degrading zonula occludens-1, claudin-1, and occludin, cell penetration, and tumour invasion were confirmed by in vitro investigations. Tumour depth and diameter were related to dentilisin expression [65]. (Fig. 2)

Other virulence factors implicated are the major outer sheath protein (MSP), the periplasmic flagella [89], and hemin-binding proteins [90]. The major outer sheath protein is present in the outer membrane vesicles of *T. denticola*. It can alter the chemotaxis signalling pathway of neutrophils, which affects the tumour suppressor function of the PTEN enzyme, facilitating tumour development [78]. MSP is pro-inflammatory through IL-6, IL-1beta, TNF-alpha, and matrix metalloprotease 9 [91]. Furthermore, it can promote cell proliferation by activating the MAPK pathway. Similarly, the flagella of *T. denticola* allow tissue penetration and mediate the immune response through the pro-inflammatory cytokines TNF-α, IL-1β, IL-6, and IL-12, or via its anti-inflammatory cytokine, IL-10 [89].

Among its virulence factors, the Leucine-rich repeat protein (LrrA) assists epithelial cell binding [92]. *T. denticola* produces H_2_S that can induce erythrocyte membrane disruption. Further, transposase-like enzyme genes in *T. denticola* can enhance virulence [93].

### Tissue presence in OSCC

This scoping review excluded studies of *T. denticola* from distant cancers [62, 94]. Xiao et al. [50]. found a higher likelihood of cancer incidence in the presence of *T. denticola*. A systematic review by Chang et al. considered only one oral carcinoma-related study, with a small sample size of six patients, yielding a low abundance of *T. denticola* and higher abundances of *Fusobacterium* and *Porphyromonas* species [95].

Three observational studies reported a higher abundance of *Treponema* (genus and species level) in the oral microbiome of saliva, tissue and swab samples in 85 head and neck carcinomas [26, 96, 97]. The study by Wolf et al. [96] had healthy control subjects but only a small number of 11 patients. The two other studies considered the adjacent relatively normal sites for comparison from the same group of cancer patients, which hinders definitive interpretation. Unlike most studies, Kaliamoorthy [28] focused on *T. denticola*. He sampled eight OSCC tissue samples, which showed the presence *T. denticola*, while no control tissue specimens had it in 30 OSCC and non-cancerous tissue samples [28].

The reviewed literature found *T. denticola* in oral carcinomas, albeit in quantities less than other prominent bacteria. Yet, it remains unclear whether this association is causal or consequential. Evidence for the carcinogenic potential of *T. denticola* is primarily from in *vitro* investigations. In their review, Beltram et al. [55] mention only one study by Sakamoto et al. on *T. denticola* [98], which investigates oral tissue damage and bacterial translocation to lymph nodes but does not mention *T. denticola* specifically. There was repeated citation of the same studies by several authors, pointing to a dearth of research on *Treponema denticola*.

### Role in oral carcinoma

**Fig. 3:**
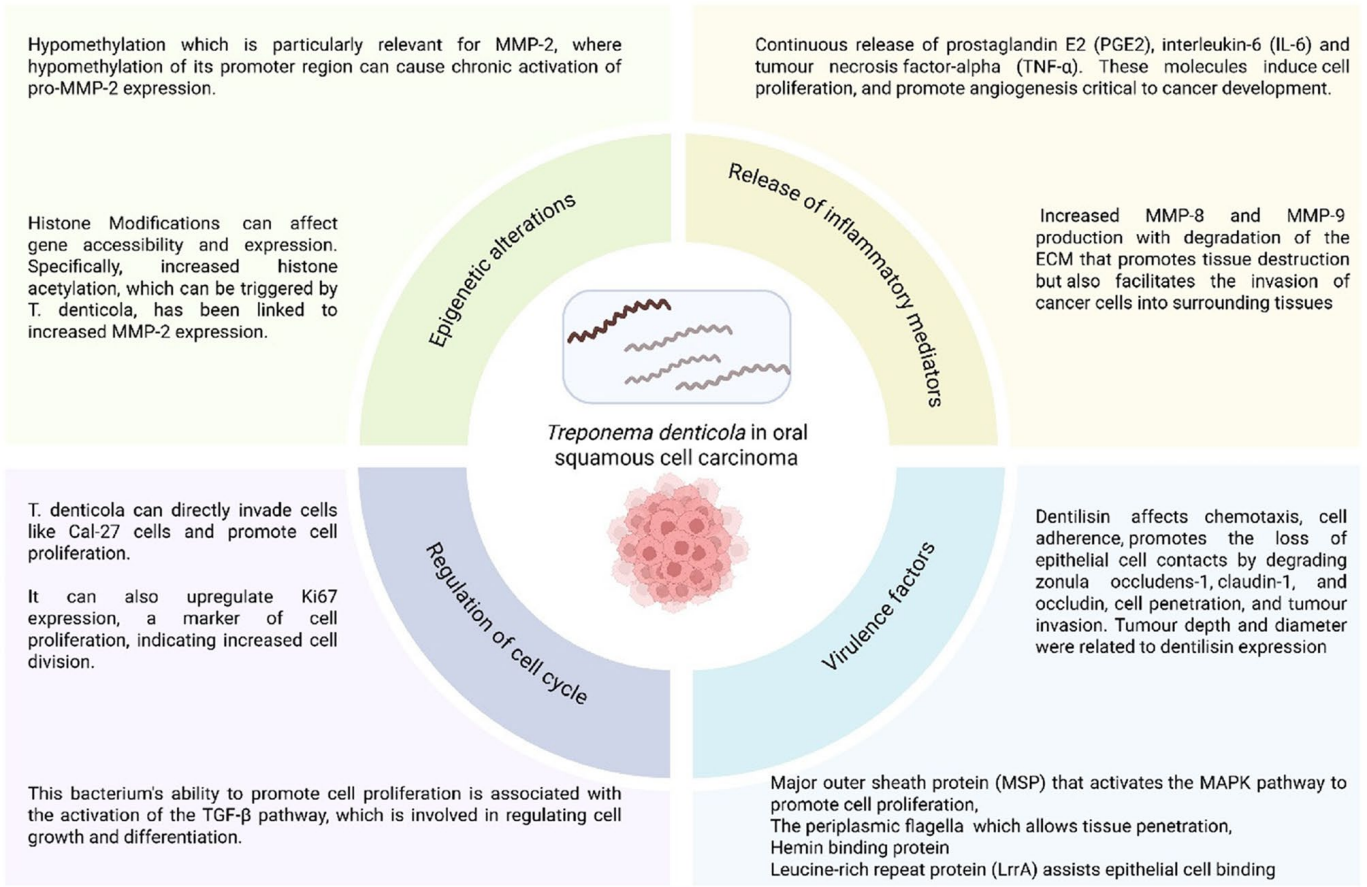
Overview of the role of *T. denticola* in OSCC

Studies have shown the direct role of *T. denticola* through tissue invasion, tumour size growth, cell proliferation, apoptosis, genetic and epigenetic alterations, and pro-inflammatory activity through proteases and reactive oxygen species. Indirect effects through its virulence factors, Td-CTLP (dentilisin), MSP and flagella also promote tumour initiation and progression. *T. denticola* regulates the cell cycle via the TGF-β pathway and inhibits cell apoptosis [65]. Kamarajan et al. [74] demonstrated the direct effects of *T. denticola* by injecting it into mice, where larger tumours were observed compared with pathogen-free control mice. We must be aware that *T. denticola* was not introduced solo but with two other pathogens, *F. nucleatum* and *P. gingivalis*. *T. denticola’s* association also emerges from the reversal of aggressive effects on tumours by probiotics like Nisin [74]. A culture model demonstrated that *T. denticola* did not invade the epithelium, but it released dentilisin in vesicles to increase epithelial permeability [99]. Several studies support this tissue invasiveness by dentilisin [100–102]. Hence, studies not exhibiting this *spirochete* could be misinterpreted as not being associated with oral carcinoma. Stealth tissue penetration is likely and can account for its carcinogenicity even in lesser quantities. Most animal model studies concentrated on periodontitis rather than carcinomas [68]. Although clinical studies do not equivocally state that *T. denticola* is critical for the pathogenesis of oral squamous cell carcinoma, in vitro studies indicate a possible link. Figure 3 gives an overview of the potential role of *T. denticola* in OSCC.

Difficulty in culturing *T. denticola* and the existence of subspecies have made studying *T. denticola* a challenge. The prevailing diagnostic methods could characterise the microbiome up to the genus level. *Treponema denticola* has been largely overshadowed by *Porphyromonas gingivalis* and *Fusobacterium nucleatum* in oral carcinoma studies [81, 103] and has not been the focus of OSCC-specific investigations. The relatively low abundance of *T. denticola* at the observed OSCC sites has belittled its stance as an oncopathogen. Current research focuses on the abundance of microorganisms observed in OSCC, which has overshadowed the contributions of organisms present in smaller quantities. Among pre-cancerous conditions, *Treponema* sp. HMT-927 was one of the five key species in oral submucous fibrosis in a study by Chen et al. [46]. In contradiction, *Treponema* species is not mentioned in seven selected studies on leucoplakia, a precancerous lesion, by another systematic review [104]. Based on the observations made, there is immense potential for further research on *Treponema denticola.*

### Strengths and limitations

Our scoping review is limited to the articles included in this search based on the given keywords, which may have excluded several other articles on *Treponema denticola* not related to our focused question. We excluded other types of oral malignancies. We could not group similar taxonomic levels, sampling strategies or detection methods. The objective of the scoping review was to note the presence of the *Treponema* genus or species. However, these dissimilarities could influence the distribution of the microbiome.

We reviewed 15 case-control studies, 16 cross-sectional studies and 4 cohort studies. Case-control studies assess multiple risk factors but are affected by recall bias. Causal associations also cannot be inferred. Cases and controls were not well-matched in all studies. Confounders such as HPV status, tooth loss, and oral hygiene status may also affect the microbiome. Periodontal parameters were also not evaluated. The inclusion of more cohort studies would have strengthened the interpretation, presenting a likely cause-and-effect relationship. Retrospective cohort studies can be impacted by observations of missing data. Most studies were cross-sectional, which has a lower reliability of evidence. Further, the scoping review did not consider information from non-English literature. Only Mendeley was used for de-duplication. No formal risk of bias was assessed, and therefore, the observations made are limited to methodological heterogeneity. The scoping review protocol has not been registered and does not need ethics approval as already existing published data is being analysed. Further, the JBI methodology framework was also followed.

Nevertheless, the studies have employed advanced molecular biology techniques for microbial detection, which strengthens their quality. Future research can investigate the functional aspects of *Treponema* species. Prospective OSCC cohorts can include studies with *Treponema* species-level investigations, a baseline periodontal examination, and adopt study models with dentilisin to demonstrate signalling of epithelial-mesenchymal transformation. Animal studies could compare *Treponema denticola* alone and in consortia with other periodontal pathogens with dentilisin-deficient models. *Treponema denticola* biomarkers from saliva or the tumour surface could be investigated for sensitivity against other bacterial panels, like *P. gingivalis* and *F. nucleatum*. Intervention studies are also needed to determine the safety endpoints of nisin or other dentilisin-targeting inhibitors.

Our paper does not suggest that a single species, in this case, *Treponema denticola*, is a cause of cancer. Cancers may be associated with mixed populations of microbes. Many microbes, including bacteria, viruses and fungi, have been implicated or suggested to contribute to the cause of cancer. The host response to mixed microbial populations that drive inflammation may explain such connections.

#### Translational relevance

Changes in the oral biofilm may impact the pathophysiology of oral carcinoma. Therefore, bacterial replacement strategies with probiotics can hypothetically provide a clinical management approach. Similarly, virulence factors can elicit a low-grade immune-inflammatory response, which is contributory to carcinogenesis. Therefore, periodontal treatment of the biofilm and control of virulence factors are rational clinical preventive avenues.

## Conclusion

The evidence maps the presence of the *Treponema* genus and species, along with dentilisin in some OSCC samples. In vitro/animal data suggest plausible roles in barrier disruption, EMT signalling, and immune evasion, often within a polymicrobial context. Validation in species-resolved, confounder-controlled human cohorts is needed before diagnostic claims.

In conclusion, the presence of *T. denticola* in the tumour microenvironment underscores the complex relationship between infection, inflammation, and cancer. *T. denticola* has been barely noticed in the oral cancer microbiome. Therefore, prospective studies can investigate its role as an oral oncopathogen. As research on the influence of the oral microbiome in oral carcinogenesis continues to expand, it is imperative to understand how pathogens like *T. denticola* will be vital for advancing prevention, diagnosis, and treatment strategies for oral squamous cell carcinoma.

## Supplementary Information

Supplementary Material 1.

Supplementary Material 2.

Supplementary Material 3.

## Data Availability

No datasets were generated or analysed during the current study.

## Abbreviations

OSCC: Oral squamous cell carcinoma
JBI: The Joanna Briggs institute
PCR: Polymerase chain reaction
RNA: Ribonucleic acid
DNA: Deoxy Ribonucleic acid
Td-CTLP (dentilisin): *Treponema denticola*, chymotrypsin like protease
HPV: Human papillomavirus
MMP: Matrix metallo protease
TCGA: The cancer genome atlas
TCMA: The cancer microbiome atlas
CRP: C-Reactive protein
