# Supplementary material for: The potential role of genus Treponema in carcinogenesis with a focus on oral squamous cell carcinoma: a scoping review of the evidence
Source: BMC Oral Health. 2025 Nov 26;26:171. doi: 10.1186/s12903-025-07118-4 (PMC12837106; doi:10.1186/s12903-025-07118-4)
Supplement: Supplementary file 1 — Supplementary Material 1. [file 12903_2025_7118_MOESM1_ESM.docx]

**Supplementary Material 1**

**Table: Characteristics of included publications**

| No. | Country | No. of papers |
| --- | --- | --- |
| 1 | USA | 19 |
| 2 | India | 11 |
| 3 | China | 13 |
| 4 | Finland | 6 |
| 5 | Korea | 3 |
| 6 | Taiwan | 2 |
| 7 | Japan | 2 |
| 8 | Sweden | 2 |
| 9 | Romania | 2 |
| 10 | Colombia | 1 |
| 11 | Iran | 1 |
| 12 | Poland | 1 |
| 13 | Australia | 1 |
| 14 | UK | 1 |
| 15 | Canada | 1 |
