# Supplementary material for: The potential role of genus Treponema in carcinogenesis with a focus on oral squamous cell carcinoma: a scoping review of the evidence
Source: BMC Oral Health. 2025 Nov 26;26:171. doi: 10.1186/s12903-025-07118-4 (PMC12837106; doi:10.1186/s12903-025-07118-4)
Supplement: Supplementary file 2 — Supplementary Material 2. [file 12903_2025_7118_MOESM2_ESM.docx]

**Supplementary Material 2**

**Terms used in the PubMed search**

((((Adult* OR patient*OR population*OR person* OR people) AND (Male* OR female*)) NOT (child*)) AND (Spirillum dentium OR Spirochaeta ambigua OR Spirochaeta comandonii OR Spirochaeta dentium OR Spirochaeta microdentium OR Spirochaeta orthodonta OR Spirochaete denticola OR Spirochaete dentium OR Spironema dentium OR Treponema ambiguum OR Treponema comandonii OR Treponema dentium OR Treponema dentium-stenogyratum OR Treponema microdentium OR Treponema orthodontum OR Treponema)) AND ((Mouth Neoplasm OR Oral Neoplasm OR Oral Neoplasms OR Cancer of the Mouth OR Oral Cancer OR Mouth Cancer OR Cancer of Mouth OR Tongue cancer OR Floor of the mouth cancer) OR (Squamous Cell Carcinoma of the Mouth OR Oral Tongue Squamous Cell Carcinoma OR Oral Cavity Squamous Cell Carcinoma OR Oropharyngeal Squamous Cell Carcinoma OR Oral Squamous Cell Carcinoma OR Oral Squamous Cell Carcinomas))OR ((epithelial-mesenchymal transition) OR (Oral cancer initiation, promotion, progression)) OR (Carcinoma)

(("adult*"[All Fields] OR ("patient*or"[All Fields] AND "population*or"[All Fields] AND "person*"[All Fields]) OR ("people s"[All Fields] OR "peopled"[All Fields] OR "peopling"[All Fields] OR "persons"[MeSH Terms] OR "persons"[All Fields] OR "people"[All Fields] OR "peoples"[All Fields])) AND ("male*"[All Fields] OR "female*"[All Fields])) NOT "child*"[All Fields] "treponema denticola"[MeSH Terms] OR ("treponema"[All Fields] AND "denticola"[All Fields]) OR "treponema denticola"[All Fields] OR ("spirillum"[All Fields] AND "dentium"[All Fields]) OR "spirillum dentium"[All Fields] treponema denticola"[MeSH Terms] OR ("treponema"[All Fields] AND "denticola"[All Fields]) OR "treponema denticola"[All Fields] OR ("spirochaeta"[All Fields] AND "ambigua"[All Fields]) OR "spirochaeta ambigua"[All Fields] ("spirochaeta"[All Fields] AND "dentium"[All Fields]) OR "spirochaeta dentium"[All Fields] ("spirochaeta"[All Fields] AND "microdentium"[All Fields]) OR "spirochaeta microdentium"[All Fields] OR ("spirochaeta"[All Fields] AND "orthodonta"[All Fields]) OR "spirochaeta orthodonta"[All Fields] OR ("spirochaete"[All Fields] AND "denticola"[All Fields]) OR "spirochaete denticola"[All Fields] OR ("treponema"[All Fields] AND "dentium"[All Fields]) OR "treponema dentium"[All Fields]OR "treponema"[MeSH Terms] OR "treponema"[All Fields] OR "treponemas"[All Fields]

"mouth neoplasms"[MeSH Terms] OR ("mouth"[All Fields] AND "neoplasms"[All Fields]) OR "mouth neoplasms"[All Fields] OR ("mouth"[All Fields] AND "neoplasm"[All Fields]) OR "mouth neoplasm"[All Fields] OR ("mouth neoplasms"[MeSH Terms] OR ("mouth"[All Fields] AND "neoplasms"[All Fields]) OR "mouth neoplasms"[All Fields] OR ("oral"[All Fields] AND "neoplasm"[All Fields]) OR "oral neoplasm"[All Fields]) OR ("mouth neoplasms"[MeSH Terms] OR ("mouth"[All Fields] AND "neoplasms"[All Fields]) OR "mouth neoplasms"[All Fields] OR ("oral"[All Fields] AND "neoplasms"[All Fields]) OR "oral neoplasms"[All Fields]) OR ("mouth neoplasms"[MeSH Terms] OR ("mouth"[All Fields] AND "neoplasms"[All Fields]) OR "mouth neoplasms"[All Fields] OR ("cancer"[All Fields] AND "mouth"[All Fields]) OR "cancer of the mouth"[All Fields]) OR ("mouth neoplasms"[MeSH Terms] OR ("mouth"[All Fields] AND "neoplasms"[All Fields]) OR "mouth neoplasms"[All Fields] OR ("oral"[All Fields] AND "cancer"[All Fields]) OR "oral cancer"[All Fields]) OR ("mouth neoplasms"[MeSH Terms] OR ("mouth"[All Fields] AND "neoplasms"[All Fields]) OR "mouth neoplasms"[All Fields] OR ("mouth"[All Fields] AND "cancer"[All Fields]) OR "mouth cancer"[All Fields]) OR ("mouth neoplasms"[MeSH Terms] OR ("mouth"[All Fields] AND "neoplasms"[All Fields]) OR "mouth neoplasms"[All Fields] OR ("cancer"[All Fields] AND "mouth"[All Fields]) OR "cancer of mouth"[All Fields]) OR ("tongue neoplasms"[MeSH Terms] OR ("tongue"[All Fields] AND "neoplasms"[All Fields]) OR "tongue neoplasms"[All Fields] OR ("tongue"[All Fields] AND "cancer"[All Fields]) OR "tongue cancer"[All Fields]) OR (("mouth floor"[MeSH Terms] OR ("mouth"[All Fields] AND "floor"[All Fields]) OR "mouth floor"[All Fields] OR ("floor"[All Fields] AND "mouth"[All Fields]) OR "floor of the mouth"[All Fields]) AND ("cancer s"[All Fields] OR "cancerated"[All Fields] OR "canceration"[All Fields] OR "cancerization"[All Fields] OR "cancerized"[All Fields] OR "cancerous"[All Fields] OR "neoplasms"[MeSH Terms] OR "neoplasms"[All Fields] OR "cancer"[All Fields] OR "cancers"[All Fields])) OR ("squamous cell carcinoma of head and neck"[MeSH Terms] OR ("squamous"[All Fields] AND "cell"[All Fields] AND "carcinoma"[All Fields] AND "head"[All Fields] AND "neck"[All Fields]) OR "squamous cell carcinoma of head and neck"[All Fields] OR ("squamous"[All Fields] AND "cell"[All Fields] AND "carcinoma"[All Fields] AND "mouth"[All Fields]) OR "squamous cell carcinoma of the mouth"[All Fields] OR ("squamous cell carcinoma of head and neck"[MeSH Terms] OR ("squamous"[All Fields] AND "cell"[All Fields] AND "carcinoma"[All Fields] AND "head"[All Fields] AND "neck"[All Fields]) OR "squamous cell carcinoma of head and neck"[All Fields] OR ("oral"[All Fields] AND "tongue"[All Fields] AND "squamous"[All Fields] AND "cell"[All Fields] AND "carcinoma"[All Fields]) OR "oral tongue squamous cell carcinoma"[All Fields]) OR ("squamous cell carcinoma of head and neck"[MeSH Terms] OR ("squamous"[All Fields] AND "cell"[All Fields] AND "carcinoma"[All Fields] AND "head"[All Fields] AND "neck"[All Fields]) OR "squamous cell carcinoma of head and neck"[All Fields] OR ("oral"[All Fields] AND "cavity"[All Fields] AND "squamous"[All Fields] AND "cell"[All Fields] AND "carcinoma"[All Fields]) OR "oral cavity squamous cell carcinoma"[All Fields]) OR ("squamous cell carcinoma of head and neck"[MeSH Terms] OR ("squamous"[All Fields] AND "cell"[All Fields] AND "carcinoma"[All Fields] AND "head"[All Fields] AND "neck"[All Fields]) OR "squamous cell carcinoma of head and neck"[All Fields] OR ("oropharyngeal"[All Fields] AND "squamous"[All Fields] AND "cell"[All Fields] AND "carcinoma"[All Fields]) OR "oropharyngeal squamous cell carcinoma"[All Fields]) OR ("squamous cell carcinoma of head and neck"[MeSH Terms] OR ("squamous"[All Fields] AND "cell"[All Fields] AND "carcinoma"[All Fields] AND "head"[All Fields] AND "neck"[All Fields]) OR "squamous cell carcinoma of head and neck"[All Fields] OR ("oral"[All Fields] AND "squamous"[All Fields] AND "cell"[All Fields] AND "carcinoma"[All Fields]) OR "oral squamous cell carcinoma"[All Fields]) OR ("squamous cell carcinoma of head and neck"[MeSH Terms] OR ("squamous"[All Fields] AND "cell"[All Fields] AND "carcinoma"[All Fields] AND "head"[All Fields] AND "neck"[All Fields]) OR "squamous cell carcinoma of head and neck"[All Fields] OR ("oral"[All Fields] AND "squamous"[All Fields] AND "cell"[All Fields] AND "carcinomas"[All Fields]) OR "oral squamous cell carcinomas"[All Fields]))"epithelial mesenchymal transition"[MeSH Terms] OR ("epithelial mesenchymal"[All Fields] AND "transition"[All Fields]) OR "epithelial mesenchymal transition"[All Fields] OR ("epithelial"[All Fields] AND "mesenchymal"[All Fields] AND "transition"[All Fields]) OR "epithelial mesenchymal transition"[All Fields] OR (("mouth neoplasms"[MeSH Terms] OR ("mouth"[All Fields] AND "neoplasms"[All Fields]) OR "mouth neoplasms"[All Fields] OR ("oral"[All Fields] AND "cancer"[All Fields]) OR "oral cancer"[All Fields]) AND ("initial"[All Fields] OR "initially"[All Fields] OR "initials"[All Fields] OR "initiate"[All Fields] OR "initiated"[All Fields] OR "initiates"[All Fields] OR "initiating"[All Fields] OR "initiation"[All Fields] OR "initiations"[All Fields] OR "initiator"[All Fields] OR "initiators"[All Fields]) AND ("promote"[All Fields] OR "promoted"[All Fields] OR "promotes"[All Fields] OR "promoting"[All Fields] OR "promotion"[All Fields] OR "promotional"[All Fields] OR "promotions"[All Fields] OR "promotive"[All Fields]) AND ("disease progression"[MeSH Terms] OR ("disease"[All Fields] AND "progression"[All Fields]) OR "disease progression"[All Fields] OR "progression"[All Fields] OR "progress"[All Fields] OR "progressed"[All Fields] OR "progresses"[All Fields] OR "progressing"[All Fields] OR "progressions"[All Fields] OR "progressive"[All Fields] OR "progressively"[All Fields] OR "progressives"[All Fields])) OR ("carcinoma"[MeSH Terms] OR "carcinoma"[All Fields] OR "carcinomas"[All Fields] OR "carcinoma s"[All Fields])
